# Burkitt's lymphoma as a pathological lead point in pediatric intussusception: A case report

**DOI:** 10.1016/j.ijscr.2024.110710

**Published:** 2024-12-01

**Authors:** Ahmed Hadj Taieb, Mohamed Ali Chaouch, Ramzi Beltaifa, Mohamed Zayati, Besma Gafsi, Faouzi Noomen

**Affiliations:** a Department of Visceral and Digestive Surgery, Monastir University Hospital, Monastir, Tunisia; b Department of Anesthesiology, Monastir University Hospital, Monastir, Tunisia

**Keywords:** Burkitt's lymphoma, Intussusception, Pediatric, Ileocolic, Case report, Surgical resection

## Abstract

**Background and importance:**

Intussusception is the invagination of one segment of the bowel into an adjacent segment, commonly causing bowel obstruction in pediatric patients. Although typically idiopathic, it can occasionally result from pathological lead points such as Burkitt's lymphoma. This case report details an unusual instance of ileocecal Burkitt's lymphoma presenting as ileocolic intussusception in a 14-year-old boy, managed surgically.

**Case presentation:**

A 14-year-old male with no significant medical history presented with right iliac fossa pain for 48 h, without fever, bowel disturbance, or vomiting. Physical examination revealed tenderness and a palpable mass in the right iliac fossa. Ultrasonography confirmed ileocolonic intussusception. Conservative treatment with ultrasound-guided hydrostatic reduction failed, leading to an abdominal CT scan, which identified an 8 cm cecal mass causing the intussusception. The patient underwent laparoscopic surgery, revealing ileocecal intussusception with multiple mesenteric lymphadenopathies. A right hemicolectomy with ileocolic anastomosis was performed. Histopathology confirmed Burkitt's lymphoma. The patient was referred for chemotherapy and recovered uneventfully postoperatively.

**Clinical discussion:**

Intussusception can occasionally be secondary to pathological conditions such as Burkitt's lymphoma. Gastrointestinal involvement in Burkitt's lymphoma is rare but can present diagnostic challenges, mimicking acute appendicitis or bowel obstruction. Diagnosis involves imaging modalities like ultrasound and CT, with tissue histopathology confirming the diagnosis. Complete surgical resection followed by chemotherapy is crucial for improved survival outcomes.

**Conclusion:**

Burkitt's lymphoma should be considered in pediatric intussusception cases, particularly when conservative management fails. Surgical intervention is essential for the diagnosis and management of intussusception caused by underlying malignancies. Early diagnosis and comprehensive treatment, including surgery and chemotherapy, are imperative for favorable outcomes.

## Highlights

• This case report details an unusual instance of ileocecal Burkitt’s lymphoma presenting as ileocolic intussusception in a pediatric patient, emphasizing the importance of considering malignancies as potential lead points in intussusception cases.
• The report underscores the diagnostic challenge posed by Burkitt’s lymphoma, mimicking symptoms of acute appendicitis or bowel obstruction, and highlights the successful use of imaging techniques and laparoscopic surgery followed by chemotherapy for effective management.
• Early and comprehensive treatment, including surgical resection and subsequent chemotherapy, is crucial for favourable outcomes in pediatric patients with intussusception caused by underlying Burkitt's lymphoma.

## 1 Introduction

Intussusception refers to the telescoping or invagination of one segment of the bowel (intussusceptum) into an adjacent segment (intussuscipiens). It represents a frequent etiology of bowel obstruction in pediatric patients. In a nationwide study in Switzerland, the mean yearly incidence of intussusception was 38, 31, and 26 cases per 100,000 live births in the first, second, and third years of life, respectively. The incidence decreases after the third year of life to less than one-half of these rates [1,2]. Intussusception is classified according to location, entero-enteric, entero-colic, and colo-colic. Ileocolic intussusception accounts for 80 % of the cases [3]. Burkitt's lymphoma, one of the causes of intussusception, was initially described in 1958 by Dennis Burkitt [4]. We report an unusual case, according to SCARE guidelines [5], of ileocecal Burkitt's lymphoma presented with right iliac fossa pain due to ileocolic intussusception.

## 2 Case presentation

**Fig. 1:**
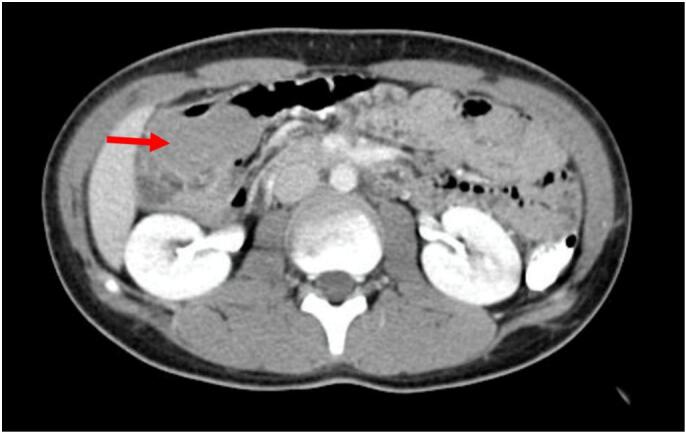
CT scan images showing the intussusception (red arrow).

**Fig. 2:**
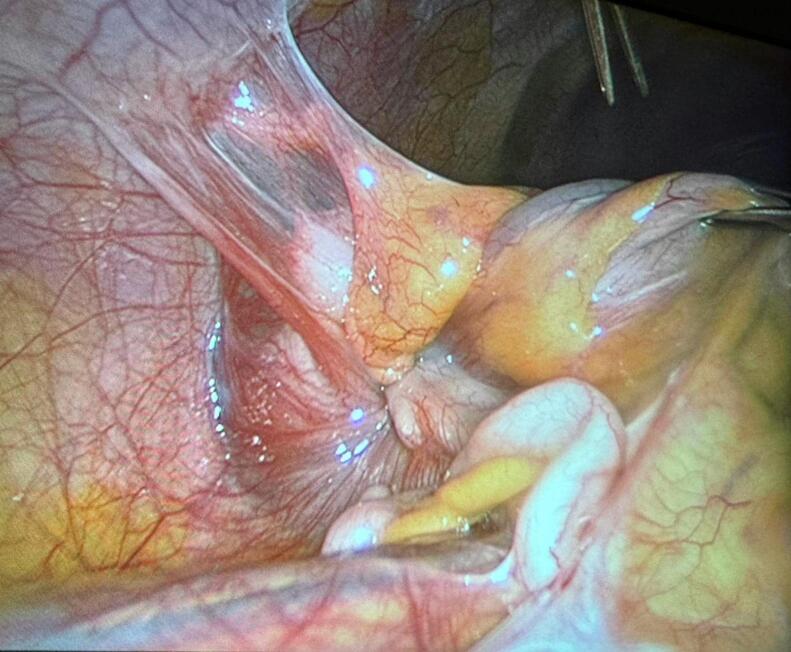
Intra operative view of the ileo-colonic intussusception.

A 14-year-old patient, with no significant medical history, presented to the Emergency department with right iliac fossa pain for 48 h, without fever, bowel disturbance, or vomiting. The patient was afebrile on clinical examination, with tenderness and a palpable mass in the right iliac fossa. At the same time, the remainder of the abdomen was nondistended, soft, depressible, and non-tender. Laboratory tests did not reveal any signs of inflammatory syndrome. Ultrasonography revealed an ileocolonic intussusception. After conservative treatment with ultrasound-guided hydrostatic reduction failed, an abdominal CT scan was performed. This CT scan aimed to assess if there was a mechanical small bowel occlusion or signs of gravity. It revealed a sub-hepatic mass referring to the ileocolonic intussusception caused by a cecal mass of 8 cm and mesenteric adenopathies (Fig. 1). Regarding the failure of the hydrostatic reduction, the patient underwent laparoscopic surgery during which an ileocecal intussusception with multiple mesenteric lymphadenopathies was found (Fig. 2). The decision was to perform a right hemicolectomy with an ileocolic anastomosis. The right colectomy was performed using the caudal-to-cranial approach. Postoperatively, the patient had an uneventful recovery. The histopathological examination of the right hemicolectomy specimen revealed an 8 cm cecal mass, consistent with Burkitt lymphoma. The tumor exhibited the characteristic “starry sky” appearance, composed of small to medium-sized lymphoid cells with high mitotic activity (>90 % Ki-67 proliferation index). Immunohistochemical analysis confirmed the diagnosis, with tumor cells expressing CD20, CD10, and BCL6, and showing MYC overexpression. Margins of resection were free of tumor involvement, and no perineural or vascular invasion was identified. Examination of the mesenteric lymph nodes revealed lymphoma involvement in 5 out of 22 nodes, confirming the regional spread of the disease. The adjacent ileum and colon appeared normal without inflammatory or neoplastic changes. The appendix showed no involvement by lymphoma. These findings confirm stage II Ann Arbor classification lymphoma localized to the ileocecal region and associated mesenteric lymph nodes. He was addressed to have chemotherapy in the Haematology Department.

## 3 Discussion

Underlying pathological causes of intussusception can be identified in 1.5–12.0 % of cases [6]. These include Meckel's diverticulum, polyps, duplications, mesentery cysts, intestinal hematoma, and lymphoma. Only 10 % of non-Hodgkin's lymphoma is confined to the gastrointestinal tract [7,8]. Phillips et al. emphasized that underlying factors such as ulcerative colitis, chronic antigenic stimulation in an immunosuppressed host, or the interaction of Epstein-Barr virus with immunosuppression, might predispose patients to develop primary colonic lymphoma. Burkitt's lymphoma affects mainly the terminal part of the ileum, the cecum, and the appendix. This is due to the high concentration of lymphoid tissue in the ileocecal junction [9] and like all lymphomas, extends into the submucosal and mucosal layer circumferentially, and manifests as a parietal digestive thickening or as a mural mass. The mural mass may sometimes invaginate into the downstream digestive segment [10]. The World Health Organization has identified three subtypes of Burkitt's lymphoma: endemic, sporadic, and immunodeficiency-associated [11]. The endemic (African) type primarily affects the maxilla and mandible, whereas the nonendemic (sporadic) type primarily affects the distal ileum, cecum, and mesentery [12]. The clinical symptoms of undiagnosed Burkitt lymphoma are quite vague, posing a significant challenge in making an accurate diagnosis. The sporadic form commonly presents with abdominal swelling as a large mesenteric, retroperitoneal, or pelvic mass, tenderness, pain, or fullness. Some patients present with symptoms of bowel obstruction secondary to ileocaecal intussusception caused by tumor growth, obstruction, or bleeding, mimicking acute appendicitis [13]. The diagnostic workup for Burkitt lymphoma, like any other form of non-Hodgkin's lymphoma, is definitive by tissue diagnosis [14]. It can be following laparotomies for appendicitis, bowel perforation, obstruction, and hemorrhage. To implement appropriate therapy, cross-sectional imaging is necessary to determine the distribution, severity, and staging [14]. Imaging techniques most often used include ultrasound, CT, PET/CT, gallium scintigraphy, and bone scintigraphy [10,14]. Ultrasound is used initially if a child presents with an abdominal or pelvic mass. CT would often follow ultrasound to allow a more global assessment for bowel and visceral involvement as well as tumor staging [14]. Surgery is required in the treatment of Burkitt's lymphoma to confirm the diagnosis and to relieve the common presenting symptoms of intestinal obstruction, abdominal mass, intussusception, or acute abdomen [15]. Complete resection is associated with improved survival [15]. Some reports demonstrated a higher survival rate (58 %- 89 %) in patients having extensive surgical resection versus for patients having only partial or incomplete resection (40 %–45 %) at 2–5 years [2,16]. Although diagnosis may be difficult, the treatment has promising outcomes as children with totally resected early stage (I or II) Burkitt lymphoma have a four-year event-free survival rate of 98 % and a four-year overall survival rate (OS) of 99 % with multiagent chemotherapy agents [17].

## 4 Conclusion

In conclusion, Burkitt's lymphoma affecting the terminal ileum can often mimic acute appendicitis clinically, posing diagnostic challenges even with investigative tools. However, laparotomy resection and anastomosis remain the gold standard treatment for intussusception caused by underlying Burkitt lymphoma. Therefore, surgeons should consider Burkitt lymphoma as a potential pathological lead point in cases of intussusception, particularly when conservative management fails.

## Author contribution

MAC, MZ, and AHT wrote the draft. SBS and WB followed up with the patient and prepared figures. FN validated the draft. All authors participated in the treatment of the patients, writing, and approving the manuscript.

## Patient consent

Written informed consent was obtained from the patient to publish this case report and accompanying images. On request, a copy of the written consent is available for review by the Editor-in-Chief of this journal.

## Ethical approval

All procedures performed in studies involving human participants were by the ethical standards of the institutional and/or national research committee and with the 1964 Helsinki Declaration and its later amendments or comparable ethical standards. Ethical clearance was not necessary as the format of this paper is a case report.

## Guarantor

Mohamed Ali Chaouch.

## Research registration number

N/A

## Funding

This research did not receive specific grants from the public, commercial or not-for-profit sectors.

## Conflict of interest statement

No conflict of interest to disclose.

## References

[1] Buettcher M., Baer G., Bonhoeffer J., Schaad U.B., Heininger U. (2007). Three-year surveillance of intussusception in children in Switzerland. Pediatrics.

[2] Daoud R., Jabra S.B., Chaouch M.A., Hassine H.B., Zayati M., Noomen F. (2024). A case report of primary pancreatic lymphoma revealed by an acute pancreatitis. Int. J. Surg. Case Rep..

[3] Baud C., Taleb-Arrada I., Eulliot J., Sevette-Bechard N., David S., Saguintaah M. (2019). Diagnostic échographique d’une invagination intestinale aiguë chez l’enfant et impact thérapeutique. J Imag Diagn Interv..

[4] Bellan C., Lazzi S., De Falco G., Nyongo A., Giordano A., Leoncini L. (2003). Burkitt’s lymphoma: new insights into molecular pathogenesis. J. Clin. Pathol..

[5] Sohrabi C., Mathew G., Maria N., Kerwan A., Franchi T., Agha R.A. (2023). The SCARE 2023 guideline: updating consensus surgical CAse REport (SCARE) guidelines. Int J Surg Lond Engl..

[6] Blakelock RT, Beasley SW. The clinical implications of non-idiopathic intussusception. Pediatr. Surg. Int. 7 déc 1998; 14(3): 163–7. 10.1007/s003830050475 9880737

[7] Herrmann R, Panahon AM, Barcos MP, Walsh D, Stutzman L. Gastrointestinal involvement in non-Hodgkin's lymphoma. Cancer 1 juill 1980; 46(1): 215–22. 10.1002/1097-0142(19800701)46:1<215::aid-cncr2820460136>3.0.co;2-6 7388763

[8] Dougaz MW, Chaouch MA, Hammami M, Achouri L, Zenaidi N, Derbel B, et al. Peritoneal changes in intestinal tuberculosis. Int. J. Infect. Dis. 1 déc 2019; 89: 110–1. 10.1016/j.ijid.2019.09.023 31586578

[9] Devita R., Towbin R.B., Towbin A.J. (2019). Burkitt lymphoma causing colocolonic intussusception. Appl. Radiol..

[10] Zayati M., Chaouch M.A., Taieb A.H., Gafsi B., Abdelwahed M.B., Noomen F. (2023). A rare case of perforated gastric lymphoma presenting a life-threatening condition: a case report. Int. J. Surg. Case Rep..

[11] Ferry J.A. (2006). Burkitt’s lymphoma: clinicopathologic features and differential diagnosis. Oncologist.

[12] Dashottar S., Sunita B.S., Singh R.K., Rana V., Suhag V., Singh A.K. (2020). A case series of unusual presentations of Burkitt’s lymphoma. J. Cancer Res. Ther..

[13] Biswas S. (2007). Report of a case of abdominal Burkitt’s lymphoma presenting as localized right iliac fossa pain mimicking acute appendicitis. Internet J Surg..

[14] Biko D.M., Anupindi S.A., Hernandez A., Kersun L., Bellah R. (2009). Childhood Burkitt lymphoma: abdominal and pelvic imaging findings. Am J Roentgenol. mai.

[15] Hoxha F.T., Hashani S.I., Krasniqi A.S., Kurshumliu F.I., Komoni D.S., Hasimja S.M. (2009). Intussusceptions as acute abdomen caused by Burkitt lymphoma: a case report. Cases J. déc.

[16] Kemeny M.M., Magrath I.T., Brennan M.F. (1982). The role of surgery in the management of American Burkitt’s lymphoma and its treatment. Ann Surg. juill.

[17] Gerrard M., Cairo M.S., Weston C., Auperin A., Pinkerton R., Lambilliote A. (2008). Excellent survival following two courses of COPAD chemotherapy in children and adolescents with resected localized B-cell non-Hodgkin’s lymphoma: results of the FAB/LMB 96 international study. Br. J. Haematol..

